# Breaking tradition: should biostatistics doctoral qualifying exams evolve to better serve our students’ ability to demonstrate readiness to conduct independent research?

**DOI:** 10.3389/fpubh.2025.1612530

**Published:** 2025-06-11

**Authors:** Scarlett L. Bellamy, Lisa M. Sullivan

**Affiliations:** Department of Biostatistics, Boston University School of Public Health, Boston, MA, United States

**Keywords:** qualifying exam, biostatistics, disparities, doctoral curriculum, equity

## Abstract

Doctoral programs in science, technology, engineering, and mathematics (STEM) education often include qualifying exams as a central component of the curriculum. While these exams are designed to assess a student’s knowledge and potential to conduct independent research as part of the culminating dissertation phase of their studies, they can also inadvertently perpetuate structural biases and barriers for underrepresented groups. Biostatistics programs have increasingly focused on efforts to address diversity. While some programs had long-standing initiatives, others began following the summer of 2020. The momentum following some of these efforts has been disrupted following the recent Supreme Court ruling around the college admissions process. In response to the Association of Schools and Programs of Public Health’s Framing the Future 2030 (ASPPH FTF2030) call to action, most specifically to “create and support inclusive and anti-racist teaching, learning, and working environments,” we propose examining the structure of the written qualifying examination to mitigate potential disparities in student success in doctoral training programs including the format of the exams, the evaluation criteria, and the support available to students as they prepare for the exam. In this paper, we briefly review the history and founding of our discipline, present data on the continuing under-representation of historically marginalized groups in our field, review the basic structure and purported purpose of the qualifying exam, and finally we propose several recommendations to address this potential structural barrier and encourage others to engage in critical reflection of their curricular requirements to assess whether they promote inclusive excellence.

## 1 Introduction: history and structure of biostatistics doctoral programs

Broadly speaking, the Ph. D. is the highest academic degree in science, technology, engineering, and mathematics (STEM) fields. A Ph. D. is typically earned after an average of 4–6 years of study, including 1–2 years of formal coursework and the remainder of time spent engaged in mentored research and often, engaged as a teaching (TA) or research assistant (RA) to develop practical teaching (TA) or research (RA) skills as part of the doctoral training. The qualifying exam (QE) is typically administered sometime between completion of the first and second year of doctoral study, and the QE usually focuses on a core set of courses. In biostatistics this often includes probability, inference, and an applied biostatistical “methods” course (minimally). Upon successful completion of the QE, students complete remaining required and elective courses as they transition to the dissertation phase, where they must independently conceptualize and carry out an original, scholarly research project. The culmination of the dissertation is characterized by both a brief oral and detailed written presentation of their work, under the guidance of a dissertation committee, consisting of faculty members with relevant methodological or applied expertise in their research area of interest.

The continued use of QEs as the major and often singular tool for assessing readiness for Ph. D. level research potentially perpetuates the problematic and divisive principles embraced by the founders of the field of biostatistics whether intentionally or unintentionally. Briefly, the Department of Biostatistics at Johns Hopkins University was founded in 1918 and is considered the oldest in the United States, while the founding of biostatistics (aka biometry) as a discipline is largely attributed to the development of a core set of fundamental statistical approaches that were largely developed between the 1880s and the 1930s. These approaches were mostly created by the following individuals: Francis Galton, Karl Pearson, R. A. Fisher, Sewell Wright, Jerzy Neyman and Egon Pearson (1). In the past decade, much has been debated after seemingly “discovering” that many of these individuals developed many of these foundational methods and concepts in support of their views around eugenics, including Galton, who is considered its founder (2). For example, one of Galton’s earliest writings establishing eugenics as a field was published in the American Journal of Sociology (3) where he first described eugenics as the “science which deals with all influences that improve the inborn qualities of a race; also, those that develop them to the utmost advantage.” We include this summary on the founding of biostatistics to add context to the discussion. Specifically, we frame the discussion of reconsidering the utility of the qualifying exam as part of the doctoral graduate curriculum as a strategy to increase inclusive excellence, which is arguably counter to the ideas of some of the founders of our discipline and field of study, thus in our view, is critical to the discussion.

## 2 Biostatistics graduate programs by the numbers

The Doctoral Initiative on Minority Attrition and Completion (DIMAC) study collected and analyzed data on attrition among underrepresented minority (URM) students in science, technology, engineering, and mathematics (STEM) in doctoral programs at 21 participating institutions where URM students were defined as any U.S. citizen or permanent residents who self-identified as Black/African American, American Indian/Alaska Native or Hispanic/Latino, enrolled in doctoral programs at those institutions. The four broad fields of study for the STEM programs were characterized as engineering, life sciences, physical and mathematical sciences and social and behavioral sciences. Among the 3,829 URM STEM doctoral students who started their programs between May 1992 and April 2005, 36% withdrew from their respective graduate programs within 7 years (engineering 36%; life sciences 31%; physical and mathematical sciences 47%; and social and behavioral sciences 33%). This is compared to a 44% completion rate within the same time period, with 20% who were still enrolled after 7 years (4). These estimates are consistent with the 10-year doctoral completion rates reported earlier from the PhD Completion Project which analyzed aggregate data from student cohorts that started their doctoral studies between academic years 1992/93 and 2003/04 at 30 U.S. institutions. Specifically, the 10-year completion rate for Black/African American students pursuing doctoral degrees in science, engineering, and mathematics was 43% compared to 56% for White students in these fields (5).

**Figure 1:**
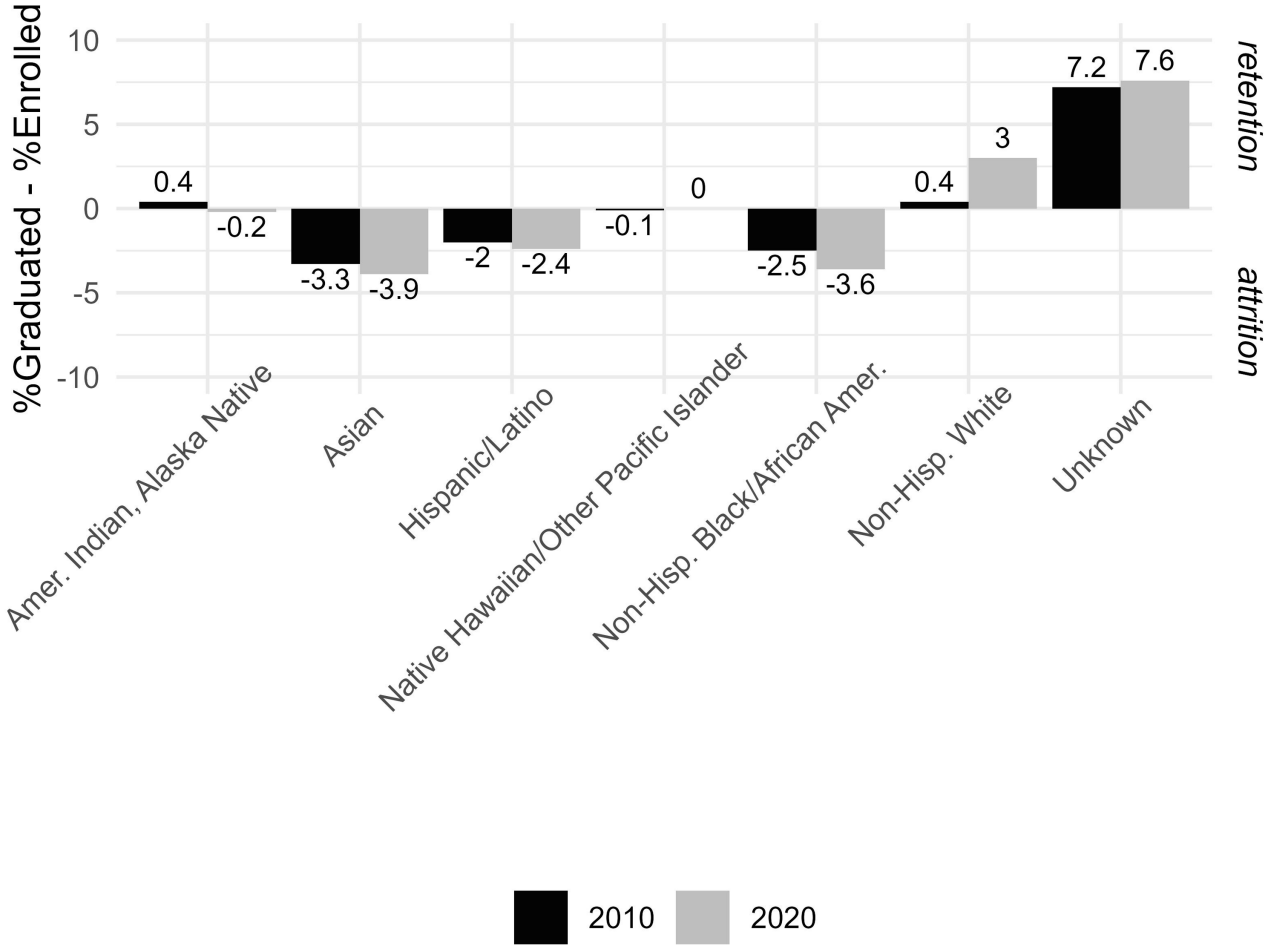
Difference in % graduates and % enrolled, among ASPPH biostatistics students in 2010 and 2020, by race/ethnicity.

There is little to no data summarizing the attrition of students who enter graduate programs in biostatistics and do not complete their graduate degrees. There is even less data published summarizing how much of that attrition is directly attributable to not successfully passing the QE. Recognizing this major limitation, we present summaries from available sources to appear in the literature. Specifically, Goodman et al. (6) summarizes student enrollments in graduate biostatistics programs as well as graduates (master’s and doctoral) of those programs using data from the Association of Schools and Programs of Public Health (ASPPH) member institutions in 2010 and 2020. Given that most students enrolled in master’s programs complete their degree within 2 years and doctoral students complete their degrees in 4–6 years, these are cross-sectional representations of these student populations for ASPPH member schools in 2010 and 2020. Neither attrition nor retention were captured for individual students, thus, as a very crude estimate of attrition, we compared the percentages of graduates and enrollees in 2010 and in 2020 as rough measure of these constructs. Here we assume that the percentages of students from each racial/ethnic group remain fairly constant. Forty-one biostatistics programs reported data to ASPPH on 514 and 635 students enrolled in biostatistics programs in 2010 and 2020 (respectively) and on 240 and 330 students who graduated in those same years. Figure 1 presents the estimated retention and attrition proxies for graduate students enrolled in biostatistics programs in 2010 (black bars) and 2020 (gray bars) computed by taking the differences in the % who graduated minus the % who were enrolled each year. When this difference is negative, it represents a proxy measure of attrition (e.g., fewer students graduate than are enrolled) and when this difference is positive, it represents a proxy measure of retention (more students graduate than are enrolled). The only groups where this difference is positive in both 2010 and 2020, indicating the percent of graduates was greater than the percent enrolled in those years (i.e., retention), are for non-Hispanic white students (0.4% in 2010 and 3.0% in 2020) or for those who did not provide their racial/ethnic identity (7.2% in 2010 and 7.6% in 2020). Differences between percentages of graduates and enrollees were consistently negative, indicating that the percent of graduates was less than the percent enrolled in both years (possible attrition), for Asian (−3.3% in 2010 and −3.9 in 2020), Hispanic/Latino (−2.0% in 2010 and −2.4 in 2020) and non-Hispanic Black/African American students (−2.5% in 2010 and −3.6 in 2020). The differences were small for American Indian/Alaska Native (0.4% in 2010 and −0.2 in 2020) and Native Hawaiian/Other Pacific Islander students (−0.1% in 2010 and 0 in 2020), indicating near steady states for these groups.

While attrition from graduate studies in biostatistics seems to be better than in other STEM fields, it remains unclear and understudied largely because of the dearth of data monitoring attrition and retention rates for doctoral students specifically.

## 3 Programmatic and structural barriers impeding equity and inclusion

Despite some recent challenges to any effort to address the chronic under-representation of some groups in biostatistics, there are a few notable, long-standing initiatives focused on addressing these barriers to entry into the profession and some have been underway for decades. Namely, efforts to diversify the field of biostatistics through a variety of training programs and workshops include the Eastern North American Region (ENAR) of the International Biometric Society’s Fostering Diversity in Biostatistics Workshop (7, 8), NIH’s Summer Institute in Biostatistics and Data Science (SIBS) initiative (9) and the Summer Program in Biostatistics and Computational Biology at Harvard (10) as well as various initiatives sponsored by the American Statistical Association (ASA) (e.g., the ASA Committee on Minorities in Statistics mentoring program at the annual Joint Statistical Meetings and StatFest (11) and the annual Women in Statistics and Data Science meeting (12), sponsored by ASA’s Women in Statistics Committee). While these efforts have been instrumental to the overall goal of making biostatistics more diverse in terms of representation, they were not designed to address the larger goal of making the profession more welcoming and inclusive. With this broader perspective in mind, the time has come to consider new, bold strategies that go beyond representative diversity.

When institutions develop initiatives to support inclusion, often what is operationalized is a form of what we term “assimilationist inclusion,” where individuals who have typically been excluded are granted entry (e.g., admissions) into those spaces, but there is little to no effort to intentionally consider how those spaces can be made more welcoming and inclusive. When those groups are considered and changes are entertained, they tend to occur when those from historically marginalized backgrounds bring their challenges to the attention of those in power and make a convincing argument to initiate change. In these contexts, there is often a perceived risk of challenging the status quo that is put on those individuals with less power and/or privilege to inform and advocate for themselves and for others, perhaps at the risk of their own academic success, even when there is no actual risk. Among other things, advocacy is often time-consuming and emotionally taxing. It is not the job of the historically marginalized to fix these structures, it is the responsibility of those in power. Yet, the spark to initiate change often rests with those groups. To be fair, there is often no ill intent, just a certain level of thoughtlessness in centering the experiences of others. In most instances, the ways of doing things have more or less been consistent since the founding of our training programs in our discipline for decades and there is a general sense that this way of doing and being is effective and is working for most, if not for all. In the spirit of making our profession welcoming and fostering belonging among those historically omitted, we suggest approaching strategies of inclusion that go beyond assimilationist inclusion, specifically and intentionally centering the perspectives of those who have historically been excluded.

## 4 Biostatistics doctoral program structure and the qualifying examination

Most graduate programs in biostatistics have a few common basic requirements, including a Bachelor’s degree where, minimally, students have had 1 year of calculus and a semester of linear algebra. While these are minimal requirements, the most attractive students have majored in a quantitative discipline, commonly mathematics or statistics, where they have had instruction in more advanced mathematics and/or statistics courses. A prior Master’s degree is required for some, but not all, PhD programs in biostatistics.

Once admitted and matriculating in a doctoral program, the basic structure of most programs can be broadly characterized as 1–2 years of formal coursework, followed by successful completion of a written qualifying examination (QE), and culminating in the production and oral defense of a dissertation. Typically, the QE exam is a comprehensive evaluation of some subset of the core curriculum covered in those first 1–2 years of graduate studies, often (minimally) covering topics in probability theory, inference and statistical methods and applications. This has been the structure for doctoral programs in STEM, including biostatistics, for nearly a century. The necessity and purpose of the coursework and dissertation phases of doctoral study are well agreed upon and articulated across various PhD programs and these details are typically well described in doctoral student handbooks. We believe there is no corresponding well-defined purpose of the QE, beyond being a necessary milestone to complete, on the way to earning one’s doctoral degree. Arguably, many feel that the QE is a necessary tool to assess whether a doctoral student has the foundational knowledge and skills required to engage in independent research (e.g., are they “qualified” to proceed to the dissertation stage pressed, would argue that preparing for the exam facilitates student’s independent synthesis of the information covered in the core courses, therefore demonstrating their mastery of this material in order to move on to the dissertation phase of their training. While we can concede that requiring a demonstration of mastery is important, we also challenge the notion that the format and structure of the QE is necessary or that it is the best tool to assess mastery and readiness to complete a dissertation. Engaging in deliberative reflection, as encouraged by FTF2030, will allow us to interrogate this notion more completely.

Further, there are no universally agreed upon competencies evaluated by the QE and typically no published rubrics detailing how student performance will be evaluated, resulting in potentially wildly different exams and outcomes both within and across institutions conferring PhDs. The fact that there is no universally agreed-upon set of competencies for the QE is problematic for at least the following reasons. First, it makes it difficult for students to know what to study, when preparing for the QE. For example, some institutions have 10 + suggested textbooks listed as references for students preparing for the QE (Table 1). And, in our collective experience, we have observed numerous cases where students earned A grades in the courses covered in the QE yet failed the QE that was designed to assess material covered in those same courses. Secondly, there is often very little feedback on performance that goes back to students, beyond whether they passed or failed the QE. Thus, for programs that do offer students a second (or third, in some instances) chance to take the exam, they often have very little guidance on exactly how they might prepare differently for their second sitting. Additionally, the breadth and depth of the courses covered by the QE can vary wildly from institution to institution.

**Table 1:** Text from online versions of doctoral student handbooks from top 10 2024 US News and World Reports *The Best Biostatistics Programs in America, Ranked* with color-coding to denote the articulated purpose and evaluation process for the doctoral qualifying examination, where available.

Program	Doctoral Handbook Text Describing Purpose and Evaluation Process of Qualifying Exam
Harvard	“The purpose of the exam is two-fold. First, it provides an opportunity for students to organize and synthesize the material covered by the four core courses; the ability to organize and synthesize a wide range of material is an important skill that the students will need as they embark on their dissertation research. Second, the exam tests the student’s understanding of probability, statistical inference, and statistical and computational methods that collectively serve as the foundation for dissertations in biostatistics. With these in mind, the written qualifying exam serves to help students achieve competencies #1 and #2. Copies of past examinations are available on request from the Senior Manager of Academic Services. The written qualifying examination is evaluated (separately) by the Qualifying Exam and Academic Standing Committees, who establish passing thresholds for the two exams. During this initial phase of the evaluation, the members of the two committees are blinded to the names of the students. In the event that one or both of a given student’s scores fall below the passing thresholds, additional evaluation is based on their performance in coursework and independent research. On the basis of this further evaluation, a student whose qualifying exam score(s) falls below the passing threshold(s) may nonetheless be determined to pass the written qualifying exam.” (21) (p. 7)
Johns Hopkins	“The Department requires a comprehensive written examination at the end of the first year (usually about 2–3 weeks after the end of fourth term) in support of student learning and as required by the Bloomberg School of Public Health. The examination consists of questions to assess competency in four core components of the program – probability, statistical theory, methods, and analysis and interpretation of data relevant to health. Students must take and pass at least three of the four components in order to pass the examination. The grading of the Departmental exam is as follows. Passing scores are determined in by exam writers after grading with examiners blinded from student names. Students who pass three of the four sections of the exam pass the exam. Students who do not pass three sections will be discussed by the faculty as a whole. This discussion will include exam and course performance. Possible resolutions include: declaring the student as passing the exam, declaring the student as having failed the exam, take-home remediation of sections of the exam or a full retake (only available if it is the student’s first attempt at the exam).” (22) (p. 17–18)
University of North Carolina-Chapel Hill	“Each PhD student is required to pass the PhD written qualifying examinations in biostatistics theory and applications. The PhD written qualifying examinations are usually taken in the beginning of the third year of the program, depending on the student’s prior obtained degree before entering the program.” (23) (p. 8).
University of Michigan-Ann Arbor	*This information is only available with login credentials from the University of Michigan*
Emory	“The written qualifying examination determines the student’s qualifications for advanced study and verifies adequate mastery of concepts in biostatistics. Students who take BIOS 512 and 513 must take the Year 1 Theory exam in the summer following enrollment in these courses. All students must take the Year 2 Methods Qualifying exams in the summer following enrollment in BIOS 522 and 709. They must also take the Year 2 Theory Qualifying exam in the summer following enrollment in BIOS 707, 710, and 711.” “Each exam question is reviewed and graded in a blinded manner by two faculty members. The results of the exams are reviewed by the graduate faculty in the Program, and a written letter with exam results is sent to each student by the Department Chair. The qualifying examination is the second component of the determination of student readiness to continue in the program. The possible outcomes of the exam are a pass, a pass with conditions, and a failure. A pass means that the student has successfully passed the exam and may now continue the process to attain candidacy. A conditional pass indicates that there are one or more areas of weakness that require additional work to be reviewed by the Examination Committee. A student receiving a failing grade may retake the examination the following year.” (24) (p. 16–17)
Columbia University	“There is a two-part qualifying examination for all PhD candidates in Biostatistics that must be completed prior to the oral comprehensive examination. The written and take-home portions of the exam are to be taken during the same summer semester.” “Grading is holistic, taking into account performance in coursework, on both portions, and other factors deemed relevant. A score below 65% on either the written or take-home portion will generally be considered unsatisfactory. The student will be allowed no more than two attempts at passing either part of the exam. It is strongly recommended that the second attempt be made at the time of the next exam offering. Exam questions from prior years are available to the student to assist in preparing for the examination. The following list consists of textbooks that are generally appropriate to use for preparing for the PhD qualifying examination.” (25) (p. 23) *Note: The handbook lists 18 textbooks as suggested references to study in preparation for the exam.*
University of Washington	“First Year Theory Exam: For advisory purposes, PhD students must take the First Year Statistical Theory Examination after the end of spring quarter following completion of STAT 512 and STAT 513 (usually in Year 1). (A new PhD student placement exam may be taken to waive these courses and the First Year Theory Exam.) • PhD Theory Exam: PhD students must pass the PhD Statistical Theory Examination within 2 years following first time completion of STAT 581, STAT 582, STAT 583 (usually the summer of Year 2). This comprehensive exam covers theory material learned in both the first and second years of the program. • PhD Applied Exam: PhD students must pass the PhD Applied Examination within 2 years following first time completion of BIOST 570 (usually the summer of Year 2). This exam covers Applied and Data Analysis coursework. In addition to courses, RA work and internships can provide opportunities to help prepare for the exam.” (26) (p.12) *Note: In addition to the exams mentioned previously, there is also a first year placement exam for doctoral students.*
University of California-Los Angeles	“Students must pass one written examination, the PH. D. Preliminary Exam. Failure to secure a passing score in at most two attempts in the PH. D. Preliminary Exam will result in the department recommending the student to the graduate division for academic disqualification. This exam is offered in September just before the beginning of fall classes. Students generally take this exam in the beginning of their second year of study. Students are expected to pass the exam at a level that would predict successful completion of the Ph. D. program. The Ph. D. Preliminary Examination covers material in the following courses and is normally taken as soon as possible after having satisfactorily completing the relevant coursework: ● Biostatistics 200 A, B and C ● Biostatistics 202 A, B Students must pass the exam at a level expected of doctoral students. Students have a maximum of two attempts to pass the exam. Students with a prior master’s degree in Biostatistics from UCLA are exempt from taking the Ph. D. Preliminary Examination, as it was taken during their MS study.” (27) (p. 24)
Boston University	“PhD candidates must satisfactorily pass two comprehensive written examinations upon completion of coursework. These require proficiency in the theory and application of biostatistics as covered in the nine core courses. Students are strongly urged to meet with their advisers to discuss preparation for the qualifying examinations. Students are allowed two attempts to pass a qualifying exam (MS or PhD). The Biostatistics Qualifying Exam Committee will evaluate requests by students to take an exam for the third time on a case-by-case basis.” *Note: The handbook lists 11 textbooks as suggested references to study in preparation for the exam; all past offerings of exams are available to students using BU credentials.* (Personal communication, October 30 (28); handbook only available with BU credentials)
University of California-Berkeley	“The primary purpose of the oral qualifying examination is to test both a candidate’s general competence in statistical theory and the ability to apply statistical methods to a subject-matter area. The exam is designed to measure breadth and depth of knowledge, as well as provide a determination of the candidate’s readiness to enter the research phase of study. *The committee for the PhD Qualifying Examination* consists of four faculty members (3 “inside” members and 1 “outside”). At least two inside members must be core SPH biostatistics faculty, one additional “inside” member must be faculty from another department but still a member of the Graduate Group in Biostatistics (for a list of core faculty and faculty who are members of the Graduate Group in Biostatistics please refer to Grad Group List), and one Academic Senate Representative (ASR), previously known as “outside” member. The ASR must belong to the UC Berkeley Academic Senate (i.e., may not be an adjunct or clinical faculty or a lecturer) and may not be a member of the Group in Biostatistics (per list mentioned above). The chair of the qualifying examination committee must be a member of the Group in Biostatistics. Additionally, *the chair of the qualifying examination committee may not serve as chair of the dissertation committee*, though it is expected that the proposed chair of the dissertation committee will serve on the qualifying examination committee. If you are earning a Designated Emphasis alongside your PhD degree (see below), you need to be admitted to that program prior to sitting for the qualifying exam and advancing to candidacy. Designated Emphases also require that you have at least one member of their faculty serve on your qualifying exam and dissertation committees. The Graduate Division must approve this committee at least 3 weeks prior to the exam itself. The candidate should meet with the chair of the qualifying examination committee to discuss the structure of the exam and any other pertinent issues.” (29) (p. 8–9).

Regarding the purpose of the QE, we started with the top 10 biostatistics graduate programs according to US News and World Reports *The Best Biostatistics Programs in America, Ranked* (13) and searched their websites for any written expression of the purpose and evaluation details of their QE (Table 1). These programs include Harvard, Johns Hopkins, the University of North Carolina-Chapel Hill, University of Michigan-Ann Arbor, Emory, Columbia University, University of Washington, University of California-Los Angeles, Boston University, and the University of California-Berkeley. While each program indicated a requirement for a written and/or oral comprehensive or qualifying examination, few provided a rationale for this requirement. Most programs outlined the details of the process and timing of the examinations, and some, but not all, programs provided minimal details on how the examinations are evaluated and scored, in such a way that would be useful to a student attempting to prepare for the exam. Despite offering few details on how the QE would be graded, more often than not, programs included exhaustive statements detailing how students would be dismissed from their doctoral program because of not successfully passing the QEs in a specified number of attempts (a maximum of two, on average). Despite the high-stakes nature of the QE, few programs offer strategies as to how students can best prepare for these exams, rubrics, competencies assessed by the exam and what, if any, supports are available to them in preparing. Common arguments in support of QEs generally center around a common theme of ensuring that students are ready to move on to conducting independent research. Specifically, many argue that the process of studying for the exam is a way for doctoral students to comprehensively review and synthesize all the material from their required courses as a way of demonstrating their readiness to conduct independent research. While we agree that the demonstration of mastery of fundamental concepts is an important step on the way to earning a doctorate, we are not convinced that the QE is the best or only way to demonstrate mastery.

We posit that these high-stakes, high-stress exams were not designed with equity or inclusion in mind and therefore, may be acting in opposition to efforts focused on increasing diversity, equity and inclusion in our graduate programs. Students enter graduate programs with varying degrees of prior preparation, based on where they received their undergraduate degrees. We do not mean this from the perspective of the quality of their undergraduate education, but rather, noting the heterogeneity of advising, mentoring, and available academic resources to support students across undergraduate institutions, including having access to advanced mathematics and statistics courses in college. The heterogeneity of student support might be even more dramatic across graduate programs as many of the highly ranked training programs in biostatistics have earned such rankings largely based on their research portfolios. In some institutions, teaching excellence may or may not be equally prioritized. The result for students in doctoral programs at those institutions may be that while there is a great benefit in working with these top researchers during the dissertation phase of study, it is unclear whether there is similar benefit to students during the earlier coursework phase of training, due to potentially misaligned incentive structures – those that value faculty teaching vs. faculty research differently.

## 5 Biostatistics training programs in the context of ASPPH’S 2030 framing the future

FTF 2030 calls for schools and programs to build inclusive excellence through an anti-racism lens in teaching, learning and working environments as a necessary step for transformation. Acknowledging the need for diversity in support of producing the most innovative scientific ideas and advances, intentional efforts to diversify the talent pool in STEM have long been established. While the pool of potential STEM graduate students is increasingly diverse, and research disciplines and institutions are striving to be more inclusive and equitable, many continue to struggle in terms of representation, climate, or both. To improve the experiences of all students enrolled in doctoral programs, the National Academies of Sciences, Engineering, and Medicine (14) proposed several recommendations in their most recent Consensus Study Report. Broadly, the report recommended that the incentive structures in academia, which have historically favored research output, should be realigned to emphasize better teaching and mentoring, and thus, a better student learning experience for graduate learners. Briefly the following were broad themes suggested in the report:

- improving STEM graduate education by adjusting faculty rewards and incentives as they pertain to teaching and mentoring;
- collecting and disseminating data to increase transparency for prospective and current STEM graduate students about institutional degree and career outcomes, among other metrics;
- increasing diversity, equity, and inclusiveness throughout STEM graduate programs to cultivate talent from all backgrounds and promote continued scientific leadership;
- building the ability of the STEM graduate education system to adjust to the dynamic nature of the scientific enterprise and the career options available to its students; and
- optimizing the experiences that graduate students have while in their programs.

The NASEM report recommends that “The graduate STEM education enterprise should enable students of all backgrounds, including but not limited to racial and ethnic background, gender, stage of life, culture, socioeconomic status, disability, sexual orientation, gender identity, and nationality, to succeed by implementing practices that create an equitable and inclusive institutional environment” (NASEM, 2018). We believe that a closer examination of all aspects of the QE experience, centering the student perspective is well aligned with the NASEM recommendations.

## 6 The qualifying exam experience from the perspective of students

For undergraduates and perhaps even Master’s degree candidates, markers of success have largely been determined by their grades in courses (via projects, exams, homework, etc.) where an earned A in a specific course, represents a certain mastery of the course material. Doctoral students also often feel successful when they earn A grades in required coursework. However, when it comes to the QE, it is entirely possible that earning straight As in the courses covered by the QE in no way assures that a student will successfully pass the QE. There are many possible reasons for this inconsistency. For example, doctoral programs rarely articulate and distribute best practices to support students as they prepare for QEs. Often, if any guidance is provided, it is usually in the form of copies of old exams, which may or may not have corresponding solutions to problems. Students preparing for the QE are often advised to “do lots of problems” in the subjects covered on the exam. Again, if students have received their undergraduate degrees at peer institutions of their graduate training programs, this kind of non-specific advice may translate well, but if they have not, it may be another form of the “hidden curriculum,” a term that is largely attributed to P. W. Jackson, that puts students from historically underrepresented backgrounds at a disadvantage (15, 16). Combined with the high-stakes nature of the QE (pass or be dismissed from your program), this is especially disturbing for graduate programs that have little transparency or that fail to center the perspectives of students (e.g., guidance on preparing, details on grading, including rubrics, what grades are required for passing, etc.). We have faculty colleagues who take great pride in writing difficult questions, well beyond the level that students have been exposed to in class, fully expecting that few, if any, students may be able to successfully produce a complete and correct solution, particularly in this anxiety-laden context. In a 2020 OpEd in the *Harvard Crimson*, O’Campo (17) gives an account of his experiences and observations during his time as a doctoral student in biostatistics at Harvard. Whether real or perceived, many of the sentiments expressed here were echoed in the piece.

Finally, while it may be well accepted that students should master a core set of fundamental concepts and theories in (bio)statistics to be able to demonstrate their readiness to take on independent research as a key component of their doctoral training, we should also recognize that upon the successful completion of the doctoral degree, rarely does one conduct independent research entirely on their own nor are we required to recall statistical information without access to a computer/book at a moment’s notice. We argue that true innovation does not happen in a vacuum and has been moving toward a team science focus as a strategy to support and accelerate discovery. This again, begs the fundamental question about the purpose of the QE and if in its current form it is the best evaluative strategy to examine readiness to participate in the scientific discovery process.

## 7 Discussion: a call to action

To be clear, we are not suggesting that standards for earning a doctoral degree in biostatistics be diminished in any respect, but rather that we reconsider how we evaluate whether or not students are sufficiently prepared to move from the coursework phase of training to the dissertation phase. We suggest that the first step to answering this question is to become clear on the purpose of the QE, beyond being an arbitrary milestone along the doctoral training continuum. Many will put forward that one of the primary objectives of the process of preparing for the QE is that it facilitates students’ independent synthesis of the information covered in the core courses evaluated by the exam. While we are not discounting the importance of this synthesis, again, we challenge the idea that this timed, high-stakes examination is the best or even the only way to achieve this broad objective of information synthesis. This is especially important as this single exam functions as a de facto “gatekeeper” for moving forward in doctoral training programs. While not directly related to the qualifying exam, there have been recent explorations of alternative evaluation approaches in statistics (18) and more broadly (19). Once the purpose can be clearly articulated and is universally accepted, a key set of minimal competencies should be established, including synthesis. Only then can the appropriate assessment be constructed. It may take the form of an exam, or it may be an entirely different process. Another notable exception is the fact that most European doctoral programs do not have a QE as part of their programs of study, with few exceptions (e.g., Sweden) (20).

We also recommend more systematic monitoring of those who do and do not demonstrate the competencies required to move to the dissertation phase, to ensure that there are not specific groups who are at higher risk. Whatever form this assessment takes, serious consideration should be given to what it fundamentally means to be a high-functioning PhD-trained biostatistician who is leading original methods research or any manner of quantitative research in a team science setting. And whatever form this assessment takes, thinking seriously about how to support all students as they prepare is essential, starting with providing rubrics for evaluation and more transparency about each step in the evaluation process.

Finally, and perhaps most importantly, if they are not already doing so, graduate programs should be systematically and regularly auditing policies and procedures to ensure that they promote inclusive excellence, and document attrition from their programs to identify if there are unexpected patterns in students who do and who do not successfully matriculate through their programs.

## Data Availability

Publicly available datasets were analyzed in this study. This data can be found at: doi: 10.1177/00333549221097653.
